# Extracellular Vesicles Loaded miRNAs as Potential Modulators Shared Between Glioblastoma, and Parkinson’s and Alzheimer’s Diseases

**DOI:** 10.3389/fncel.2020.590034

**Published:** 2020-11-04

**Authors:** Laura Thomas, Tullio Florio, Carolina Perez-Castro

**Affiliations:** ^1^Instituto de Investigación en Biomedicina de Buenos Aires – Consejo Nacional de Investigaciones Científicas y Técnicas – Partner Institute of the Max Planck Society, Buenos Aires, Argentina; ^2^Sezione di Farmacologia, Dipartimento di Medicina Interna and Centro di Eccellenza per la Ricerca Biomedica, Università di Genova, Genova, Italy; ^3^IRCCS Ospedale Policlinico San Martino, Genova, Italy

**Keywords:** miRNAs, extracellular vesicles, glioblastoma, Alzheimer’s disease, Parkinson’s disease

## Abstract

Glioblastoma (GBM) is the deadliest brain tumor. Its poor prognosis is due to cell heterogeneity, invasiveness, and high vascularization that impede an efficient therapeutic approach. In the past few years, several molecular links connecting GBM to neurodegenerative diseases (NDDs) were identified at preclinical and clinical level. In particular, giving the increasing critical role that epigenetic alterations play in both GBM and NDDs, we deeply analyzed the role of miRNAs, small non-coding RNAs acting epigenetic modulators in several key biological processes. Specific miRNAs, transported by extracellular vesicles (EVs), act as intercellular communication signals in both diseases. In this way, miRNA-loaded EVs modulate GBM tumorigenesis, as they spread oncogenic signaling within brain parenchyma, and control the aggregation of neurotoxic protein (Tau, Aβ-amyloid peptide, and α-synuclein) in NDDs. In this review, we highlight the most promising miRNAs linking GBM and NDDs playing a significant pathogenic role in both diseases.

## Introduction

Glioblastoma recapitulates most of the hallmarks frequently described in cancer, such as uncontrolled proliferation, apoptosis resistance, dysregulated cell cycle, and angiogenesis. In contrast, the opposite phenomena are described for NDDs as cell death and tissue degeneration. Therefore, a contrasting nexus between these illnesses can be inferred from this comparison. In support, cancer and NDDs have shown inverse comorbidity (at least for certain types of cancer), revealed by epidemiological studies (Driver, 2014). Indeed, several proteins such as p53, cyclin F, among others, were found to be inversely correlated in NDD with cancer. In addition, several other factors were up- or down-regulated in both diseases, including protein phosphatase 2 (PP2A), cyclins D and E, among others (Seo and Park, 2019). The existence of overlapping regulation of pathogenic molecules involved in both GBM and NDDs, although the underlying mechanisms of regulation could be distinct, raise the possibility that these molecular links may have the relevant potentiality to be exploited for developing prognostic biomarkers and therapeutic strategies. Among these linking molecules, both diseases closely associate with miRNAs up- or down-regulation that modify protein expression within the cells but can also be secreted to impact on distant cell biology, making interesting to investigate their correlations. This review analyses the biological significance of the most dysregulated miRNAs detected in GBM as molecular linkers to NDDs.

### Glioblastoma

Glioblastoma is the deadliest and one of the more frequent CNS tumor in adults. It has a poor prognosis due to its cellular heterogeneity and complexity, the high local invasiveness and the prominent neovascularization, which impede an efficient therapeutic approach. Even when surgical removal could be obtained, the tumor will most certainly relapse more aggressively, and become lethal within a year. Furthermore, different driver mutations would generate a particular microenvironment affecting GBM (Chen and Hambardzumyan, 2018), with the current cytotoxic therapies that also contribute to generate additional mutations influencing the dynamic evolution of the tumor growth and its heterogeneity (Sottoriva et al., 2013; Wang et al., 2016; Garnier et al., 2018).

From a molecular point of view, GBM can be classified into classical, mesenchymal, proneural, and neural subtypes (Verhaak et al., 2010). The most prominent marker for each subtype is EGFR, Neurofibromatosis type 1 (NF1), and PDGFRA/IDH1 genes, respectively (Verhaak et al., 2010; Galatro et al., 2017). These tumors are vastly heterogeneous and therefore, they often develop different mutations at the same time. In fact, the majority of patients show areas characterizing different GBM subtypes within the same tumor, with coexisting multiple tumor cell clones presenting different genetic and epigenetic alterations (del Inda et al., 2014; Teng et al., 2017; Neftel et al., 2019). Most of this diversity is generated as a result of the interaction between tumor cells and different environments during the growth of the tumor (Sottoriva et al., 2013; Osuka and Van Meir, 2017).

Glioblastoma is enriched in stem-like tumor cells (GSCs) (Persano et al., 2011). These cells can self-renew, and differentiate into multiple cell types, increasing the dynamic of tumor heterogeneity and resistance to drug and radiation (Chen et al., 2012; Gimple et al., 2019). GSCs usually express markers, such as CD133, a normal NSC marker in development, or CD44, a progenitor cell marker, which interaction with hyaluronic acid in extracellular matrix, favors GSC invasion of the brain (Bradshaw et al., 2016). In this regard, CD133 or CD44 expression was proposed as a criterion for GBM classification: CD133 is highly expressed in proneural GBMs and CD44 in mesenchymal GBM subtypes. Both markers are mutually exclusive and independent of the stem cells’ properties within the tumor (Brown et al., 2015). Also, GSCs express stem cell transcription factors including NANOG, SOX2, POU1F5/OCT4, c-Myc, KANSL2, POU3F2, SALL2 among others, which are involved in proliferation and pluripotency maintenance (Suvà et al., 2014; Ferreyra Solari et al., 2016).

The surroundings where GSCs home are called tumor niches and consist of heterogeneous microenvironments. They differ from the intratumor heterogeneity derived from diverse cell types and mutations. Particularly, these niches contain tumor-associated microglia/macrophages (TAMs) (Sørensen et al., 2018), which can exhibit a wide range of functional properties, displaying characteristics of either M1 or M2 microglia (in the M1/M2 model), but also in between, hence this model becomes rather too simplistic for describing TAMs. GSCs are the preferred candidates to recruit TAMs into the tumor growing mass, as they secrete several chemoattractant molecules like Interleukin (IL) 6, IL1β, VEGF, colony stimulating factor 1 (CSF1), CXCL12, and are able to induce their polarization into a pro-angiogenic and immunosuppressive phenotype, which favors tumor growth and escape from the immune surveillance (Lisi et al., 2017; Manini et al., 2018).

### Neurodegenerative Diseases

Neurodegenerative diseases are a group of illnesses characterized by progressive functional damage of neurons leading toward neuropsychiatric symptoms culminating with high cognitive impairment. For example, neuronal damage can be caused by amyloid-like protein deposition, genetic alterations, or by secondary origins like immune diseases, or metabolic dysregulations. Most of the NDDs are classified as “proteinopathies,” since they are caused by misfolded protein deposition, with the neurotoxic entity represented by the protein tridimensional structure independently by protein sequence or function. Normally these abnormal protein aggregates are scavenged by autophagy-lysosome or by the ubiquitin-proteasome system, mechanisms that demand high energy consumption and can provoke mitochondrial dysfunction and free-radical production. This oxidative stress leads to DNA damage and microglia activation, neuroinflammation, and dysfunction of neuronal transport, culminating with axonal degeneration and neuronal death (Kovacs, 2016). In this regard, the misfolded protein effects are extensively investigated for therapeutic strategies and as biomarkers.

Alzheimer’s disease is characterized by the deposition of Aβ fibrils in brain parenchyma and vessel walls, associated to the accumulation of aberrant phosphorylated Tau protein in neurons. This disease is the main cause of dementia worldwide and its etiology involves both genetic and environmental components. The pathology mostly originates as sporadic form, but can also occur from mutations in the APP gene (the precursor for Aβ fibrils), in addition to presenilin (PSEN) 1 and PSEN2 that encode a γ-secretase responsible for APP cleavage. Also, the expression of the apolipoprotein E variant, ApoE4, represents a risk factor for developing AD (Lane et al., 2018). These protein depositions in the extracellular space, mainly in the isocortex, are called amyloid plaques and are mostly composed of abnormally folded Aβ subproducts of APP metabolism. Microglia activation by Aβ oligomers increases the expression of triggering receptor in myeloid cells 2 (TREM-2) and stimulates astrocytes leading to inflammation and neuronal damage.

Parkinson’s disease (PD) is an α-synucleinopathy characterized by the formation of Lewy bodies, predominantly in neurons, along with the loss of dopaminergic neurons in the *substantia nigra pars compacta*. These neurons regulate the activity of the striatum, responsible for motor responses, thus patients develop muscle rigidity, bradykinesia, postural instability, and resting tremor. The family or hereditary form of these diseases accounts for 10 to 15% of all cases. These patients present mutations in some genes, such as *SNCA*, which encodes α-synuclein associated with the progression and the severity of the disease (Dehay et al., 2017). However, the sporadic form is the most prevalent, and the etiology remains still unknown. It was proposed that a combination of genetic susceptibility, diet, environmental influences, viral infections, among others, might act together to the onset of PD.

### miRNAs

miRNAs belong to a group of small non-coding (nc) RNAs, consisting of single-stranded RNAs of 20–24 nucleotides, that assemble into ribonucleoprotein complexes called RNA-induced silencing complex (RISC). For details, please refer to Hammond (2015) and O’Brien et al. (2020). miRNAs regulate protein levels without changing DNA sequence. In particular, a group of miRNAs called Epi-miRNAs, can modulate the expression of epigenetic machinery proteins (Moutinho and Esteller, 2017). In addition, miRNAs can also bind to a nascent mRNA, affecting transcription (Moutinho and Esteller, 2017). All of these functions categorize miRNAs as part of epigenetic mechanisms. Therefore, any deregulation of miRNAs production affects gene expression leading to several diseases including cancer (Baumann and Winkler, 2014).

### Extracellular Vesicles

**FIGURE 1:**
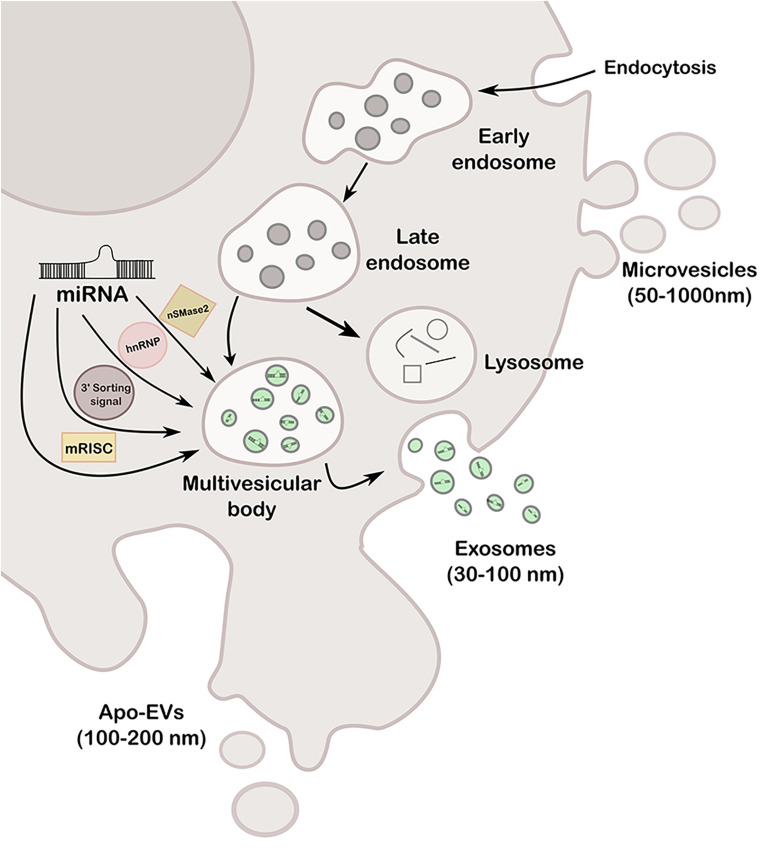
Schematic representation of the different types of EVs (exosomes, microvesicles and apo-EVs), its formation mechanisms and miRNA loading pathways (nSMase2, hnRNP, 3′ sorting signal and mRISC).

Extracellular vesicles are cell-derived particles that vary in size and synthesis and carry several cargos like RNA, DNA, lipid, and proteins, among others, of the cell of origin to a recipient cell (Rahbarghazi et al., 2019). Hence, EVs represent a novel type of cell-to-cell communication mechanism having potential capability of modifying expression and behavior of the recipient cell (Loghlen, 2017; Nogués et al., 2018; Willms et al., 2018). Depending on size and origin, EVs can be classified at least in three types: exosomes (30–100 nm), microvesicles or ectosomes (50–1000 nm), and apoptotic bodies (100–200 nm) (Wang M. et al., 2019). Exosomes are formed in a late endosomal intracellular compartment called the multivesicular body (MVB), arisen from an early endosome during the budding of the membrane, generating intraluminal vesicles. Then, MVB can either fuse with lysosomes for degradation or with the plasma membrane to release the vesicles, called exosomes, into the extracellular space. The transported exosomes can be then recognized by the interaction of its transmembrane proteins with the receptors of recipient cells, or alternatively, fuse directly to the plasma membrane, by both clathrin-dependent and independent pathways (Zhang J. et al., 2015). Microvesicles are larger than exosomes and arise directly from outward budding of the plasma membrane, generating vesicles toward the extracellular space. Regarding apoptotic bodies [known as apoptotic cell-derived extracellular vesicles (ApoEVs)], they are produced by dying cells and therefore contain organelles and cellular material that are destined to be degraded by phagocytosis (You and Ikezu, 2019). More recently, it was reported that ApoEVs are also able to transmit biological messages from the cells from which they are originated to nearby cells, and not simply garbage bags, as previously believed (Xu X. et al., 2019). The three types of vesicles are represented in Figure 1.

The most studied EVs are exosomes as they are secreted by almost every cell type. They are modulators of several physiological responses like immune responses, tissue repair, and blood coagulation, among others (Arenaccio and Federico, 2017). Particularly, they participate in tumorigenesis, for example, by spreading from cell to cell oncogenic signaling such as mutant EGFR. Besides, they are also involved in neurological disorders, as they can load and transport several proteins involved in neuropathies including Tau, Aβ-amyloid peptide, and α-synuclein (Ageta and Tsuchida, 2019).

Remarkably, the most relevant connection between exosomes and epigenetics are miRNAs since they can be loaded into exosomes by the recognition of specific sequences called EXOmotifs by one of four possible pathways, represented in Figure 1: (a) the neural sphingomyelinase 2 (nSMase2)-dependent pathway, (b) the sumoylated heterogeneous nuclear ribonucleoprotein (hnRNP)-dependent pathway, (c) the sorting signal encoded in the 3′ end of the miRNA, and (d) miRNA-induced silencing complex (miRISC) (Rahbarghazi et al., 2019). Any dysregulation of these pathways may lead to aberrant loading and a potentially consequent disease progression; therefore, these pathways could be exploited for therapy designs. miRNA-containing-exosomes are unloaded by the receptor cell where they play a role as negative regulators of transcription and expression, or instead, function as receptors for immune cells by acting like a toll-like receptor (TRL) receptor (Fabbri et al., 2012), among others.

### Relationship Between Molecular Pathways Dysregulated in GBM and PD, and AD

There is increasing evidence showing brain cancer and neurodegeneration can mutually influence each other. For example, tumor cells secrete glutamate, which drives neurotoxicity and neuronal death, and promotes tumor-associated neurodegeneration (Seo and Park, 2019). Also, tumor cells can communicate with and regulate microenvironment functioning through several mechanisms, including EVs and signaling molecules. In NDDs, cells also interact to modulate the microenvironment; for example, microglia is able to secrete pro-inflammatory factors via EVs, while astrocytes secrete EVs that drive apoptosis, generating changes in the microenvironment (for details refer to Martins et al., 2020). Therefore, modification of the microenvironment appears to be an important shared factor for the progression of both cancer and NDDs. Indeed, this intercellular communication may explain the inverse comorbidity between cancer and NDDs: low probability to develop cancer in patients with certain CNS diseases, and vice versa (Tabarés-seisdedos and Rubenstein, 2013; Ibáñez et al., 2014). In support, several epidemiological studies reported an inverse risk between the development of PD or AD and cancer (Driver, 2014). In molecular terms, both cancer and NDDs share signaling molecules but the underlying mechanisms could be distinct (Seo and Park, 2019): cancers become immortal while neurodegeneration kill cells. Therefore, it has become relevant to investigate the linking signaling molecules identified in GBM that also affect NDDs, to better understand the pathogenesis of these diseases.

Cancer and NDDs might share commonalities. Foremost, neurons are affected in both diseases. In cancer the machinery to repair DNA is frequently found mutated leading to genomic instability. In the case of NDDs, during the reentry of neurons into the cell cycle, similar mutations of the DNA repair machinery can lead to protein accumulation, tangles, and formation of plaques and ultimately to cell death (Houck et al., 2019). For example, p53, the guardian of the genome, is a potential molecular link between both diseases: p53 is often mutated in cancer and active in NDDs (Driver, 2014). In relation with miRNAs, miRNA-21 found in exosomes (Jesionek-kupnicka et al., 2017; de Mooij et al., 2020) and −10b (Sun et al., 2019) were linked to p53 in GBM. Interestingly, when the antago-miRNAs were packed in nanoparticles and used for GBM treatment, they decreased the tumor mass and led to cell cycle arrest (Teplyuk et al., 2016). In NDDs, these miRNAs are mostly linked to inflammation, and therefore the role of p53 pathways in NDDs remains further to be evaluated.

Other potential links that play a significant role in the development of both diseases are aging, oxidative stress, and inflammation (Houck et al., 2019). For example, miRNAs-23a (Li et al., 2020), −137 (Jiang Y. et al., 2019), −139 (Tang et al., 2017) were linked to oxidative stress and inflammation. Other potential commonalities between cancer and NDDs worth examining are the nutrient-sensing mechanisms and cellular senescence, which are related to aging and metabolism deregulation. Cancer cells redirect their metabolism to increase proliferation by fermenting glucose into lactate even when mitochondria are fully active, a process known as the Warburg effect (Liberti and Locasale, 2016). In NDDs, neurons are dependent on the metabolic support provided by glial cells, which can become senescent with aging (Houck et al., 2019). AD patients are often hyperglycemic and have glucose metabolism disorders such as diabetes, leading to a decreased utilization of glucose in affected regions of the brain through disease progression (Driver, 2014). In this regard, miRNA-7 was linked to glucose metabolism in AD where it targets Insulin Receptor Substrate-2 and Insulin Degrading Enzyme (Fernández-de Frutos et al., 2019). Similarly, miRNA-338 contributes to metabolic deregulation as a promoter of glycogen accumulation (Li C. et al., 2019). In line with these findings, it is also frequent to detect mitochondrial dysfunction related to energetic dysregulation in NDDs and cancer, possibly as a consequence of mitochondrial DNA mutations (Houck et al., 2019). In this context, miRNA-137 controls mitochondria biogenesis and dynamics in NSCs, leading to neuronal differentiation (Channakkar et al., 2020), and also was linked to vesicle trafficking and exosomes in PD (Jiang Y. et al., 2019); miRNA-210 was described to target COX 10 and ISCU 1/2, linked to mitochondrial dysfunction (Li et al., 2014).

Proteostasis should be also evaluated since protein processing is critical for both diseases. In cancer, the ubiquitin-proteasome system is upregulated to control the amount of proteins needed to proliferate; in contrast, the opposite occurs in NDDs (Houck et al., 2019). In PD, the ubiquitin-proteasome system is downregulated leading to α-synuclein aggregation into Lewy bodies. In AD, proteostasis is unbalanced because of mutations and dysregulation of chaperones (miRNA-210 targets P4HB chaperone; Lee et al., 2015) and autophagy, leading to Aβ-amyloid peptides and phosphorylated Tau deposits. In this regard, Tau would become a potential nexus between GBM and NDDs, as Tau is a contributor for GBM-associated neurodegeneration, altogether with mostly upregulated miRNAs −27a, −138, −195, −132, and −125 (Frigerio et al., 2013; Dehghani et al., 2018; El Fatimy et al., 2018). As mentioned above, AD is characterized by the deposition of Aβ fibrils in brain parenchyma and vessels’ wall and by the accumulation of aberrant phosphorylated Tau protein in neurons (Gargini et al., 2019). In GBM, secreted CD44 leads to hyperphosphorylation of Tau in the hippocampus (Lim et al., 2018); furthermore, besides this neurodegenerative property, Tau was implicated in GBM cells spreading: Tau expressed in GBM cells increases their motility by modulating cytoskeleton dynamics in a Rho-associated protein kinase (ROCK) signaling pathway-dependent manner, it stabilizes microtubules, and generates a rearrangement of the location of ROCK signaling pathway molecules, leading to active actin cytoskeleton dynamics and motility (Breuzard et al., 2019). Controversially, a recent *in silico* analysis revealed that Tau expression is inversely correlated with glioma progression: gliomas with higher levels of Tau have a better prognosis (Gargini et al., 2019). In line with this evidence, Tau was also found inversely correlated with glioma progression and positively correlated with IDH mutation (Gargini et al., 2020). Mechanistically, the increased expression of Tau in IDH mutated gliomas impedes EGFR signaling and the transdifferentiation into the more aggressive mesenchymal subtype. Pin1 is also related to proteostasis, and is downregulated in AD and upregulated in PD and cancer (Seo and Park, 2019). Pin1 is a G1 checkpoint controller that can bind to Tau, enhancing its dephosphorylation, and can also interact with α-synuclein to promote its aggregation (Seo and Park, 2019). Notably, upregulated proteins in NDDs were found structurally disordered, showing a clear evidence of proteinopathy. In contrast, upregulated proteins found in cancer were structurally normal, indicating that these proteins functions are normal during the development of tumor (Klus et al., 2015). Furthermore, genes upregulated in NDDs were usually less active in the normal physiological state. On the contrary, genes upregulated in cancer are mostly already very active in normal cells (Klus et al., 2015).

WNT is another interesting point to consider in tumor-associated neurodegeneration, as cancer cells steal WNT factor from normal neurons in the brain generating its death (Portela et al., 2019). WNT pathway also drives NSC proliferation, so it makes sense that the WNT pathway is downregulated in NDDs and upregulated in cancer. Concordantly, miRNAs −21 (Luo et al., 2017) and −338 (Li et al., 2018) are deeply related to WNT pathway and, upregulated and downregulated in GBM, respectively. Interestingly, miRNA-338 sponge causes spontaneous neoplasm formation (Howe et al., 2017). In NDDs, these miRNAs are mainly related to protein deposits.

In the next sections, we will highlight the most commonly deregulated miRNAs identified in both GBM and NDDs (in particular AD and PD), discussing the up-to-date knowledge of EVs and their potential link with their etiology.

## EVs – miRNAs in the Regulation of GBM

**FIGURE 2:**
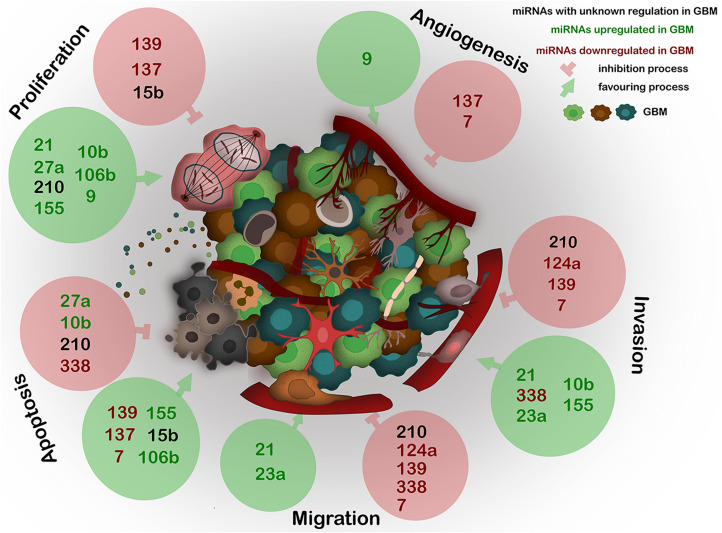
Expression of the most deregulated miRNAs in GBM and their influence on proliferation, angiogenesis, invasion, migration, and apoptosis of GBM. miRNAs, frequently carried by EVs, act as epigenetic modulators and regulate several key processes in GBM. The deregulation of any of these miRNAs generates abnormal gene expression which leads to disease progression. Black – miRNAs with unknown regulation in GBM; green – miRNAs upregulated in GBM; red – miRNAs downregulated in GBM. Green circles show miRNAs positive effect on described processes; red circles show miRNAs negative effect on described processes.

A recent major advance in GBM research was the discovery of a communication between the tumor cells and the stroma, mainly by EVs. One of the main players within the EVs are the miRNAs as they constitute a repertoire that depends on the cell of origin, and can regulate angiogenesis, proliferation, invasion, and metastasis (Ingenito et al., 2019) (Figure 2).

Several approaches were used to create a miRNA profile to classify them in patterns corresponding to different GBM histotypes. For example, exosomes derived from GBM patients’ sera were analyzed to generate a list of novel miRNAs that would be classifiers for gliomas grade II-III, IDH mutant, and GBM IDH wt. Following this approach, the authors reported that miRNAs 485, 486, and 543 were enriched in lower-grade gliomas and miRNAs 182, 328, 339, and 340 behaved as stable markers for GBM (Ebrahimkhani et al., 2018). Moreover, the ExoCarta database^1^ provides information about exosomes from diverse specimens, including miRNAs cargos and GBM samples. Similarly, a database dedicated exclusively to miRNAs^2^, enlists precursors and mature miRNA from several species. Furthermore, a dataset dedicated to GBM is available in the Cancer Genome Atlas (TCGA)^3^ and in Gene Expression Omnibus (GEO)^4^.

Recently, it was developed an analysis of GEO database specific for miRNAs involved in GBM and AD (Candido et al., 2019); the authors scrutinized the datasets and provided an *in silico* list of inversely regulated miRNAs and an ontological analysis of the data. Also, a new database was built integrating several features of GBM including genomic variants, gene and miRNA expression in patients, DNA methylation profile, among others^5^ (Yang et al., 2019a). It is important to note that in some of these databases, the associations of some miRNAs with EVs were not reported; therefore, it would become a priority to identify and classify these miRNAs to have a better perspective of the EVs-miRNAs role in these pathologies, as for some of them, EVs-miRNAs are very much unexplored.

The next sections will focus on the list of the most dysregulated miRNAs in GBM created by Bhaskaran et al. (2019), which is based on the TCGA database, summarizing the up-to-date knowledge about these miRNAs related to GBM biology, followed by potential association with EVs. Furthermore, we evaluate the possible links between these dysregulated miRNAs with AD and PD, to determine potential molecular links for these diseases (Driver, 2014). The information of the next section is summarized in Figure 2 and Table 1.

**TABLE 1:** Expression of the most deregulated miRNAs in GBM described in this work, their influence on proliferation, angiogenesis, invasion, migration, and apoptosis and their regulation in PD and AD.

miRNA	GBM						Neurodegenerative diseases: regulation		Transported by EVs
	Regulation	Proliferation	Angiogenesis	Migration	Invasion	Apoptosis	PD	AD	
21	Up (Luo et al., 2017)	+ (Jesionek-kupnicka et al., 2017)		+ (Luo et al., 2017)	+ (Luo et al., 2017)		Up (Su et al., 2016)	Down (Juźwik et al., 2019)	Yes (Van Der Vos et al., 2016; Fernandes et al., 2018; Guo et al., 2019; Xiong et al., 2019; de Mooij et al., 2020)
27a	Up (Bhaskaran et al., 2019)	+ (Ge et al., 2013)				- (Ge et al., 2013; Rivera-Díaz et al., 2015)	Down (dos Santos et al., 2018)	Down (Frigerio et al., 2013)	Yes (dos Santos et al., 2018; Liu et al., 2018; Wang J. et al., 2018)
23a	Up (Bhaskaran et al., 2019)			+ (Lian et al., 2013)	+ (Hu et al., 2013; Yachi et al., 2018)		Up (Barbagallo et al., 2019)	Down (Agostini et al., 2019; Barbagallo et al., 2019)	Yes (Hsu et al., 2017; Liu J. et al., 2019)
210	Up (Lai et al., 2015; Rosenberg et al., 2015; Zhang S. et al., 2018)/Down (Liu S. et al., 2019)	+ (Zhang S. et al., 2018)		- (Liu S. et al., 2019)	- (Liu S. et al., 2019)	- (Zhang S. et al., 2018)	Up (Zhang S. et al., 2018)	Up/Down (Li et al., 2014; Zhu et al., 2015; Swarbrick et al., 2019)	Yes (Jung et al., 2017; Lin et al., 2018; Zhang et al., 2019; Tabibkhooei et al., 2020)
10b	Up (Bhaskaran et al., 2019)	+ (Teplyuk et al., 2015)			+ (Bhaskaran et al., 2019)	- (Gabriely et al., 2011; Sun et al., 2019)	Down (Hoss et al., 2016)	Up (Lau et al., 2013)	Yes (Singh et al., 2014; Liu et al., 2017; Zhou et al., 2017; Jiang H. et al., 2019)
155	Up (Bhaskaran et al., 2019)	+ (Zhou et al., 2013)			+ (Liu et al., 2015)	+ (Liu et al., 2015)	Up (Sancandi et al., 2020)	Down (Slota and Booth, 2019)	Yes (Sancandi et al., 2020)
106b	Up (Bhaskaran et al., 2019)	+ (Liu et al., 2014)				- (Liu et al., 2014)	*	Up (Martins et al., 2020)	Yes (Ebrahimkhani et al., 2018)
15b	Up (Xia et al., 2009; Bhaskaran et al., 2019)/Down (Wang et al., 2017; Matos et al., 2018)	- (Xia et al., 2009)				+ (Wang et al., 2017)	*	Down (Martins et al., 2020)	Yes (Martins et al., 2020)
124a	Down (Fowler et al., 2011)			- (Soldati et al., 2013; Tivnan et al., 2014)	- (Soldati et al., 2013; Tivnan et al., 2014)		Down (Saraiva et al., 2016)	Down (Zhang et al., 2017a)	Yes (Morel et al., 2013; Lang et al., 2018; Yang et al., 2019b)
139	Down (Bhaskaran et al., 2019)	- (Dai et al., 2015)		- (Yue et al., 2015; Chen et al., 2018; Tian et al., 2019)	- (Yue et al., 2015; Chen et al., 2018; Tian et al., 2019)	+ (Chen et al., 2018; Tian et al., 2019)	*	Up (Tang et al., 2017)	Yes (Xu G. et al., 2019; Wei et al., 2020)
137	Down (Bhaskaran et al., 2019)	- (Sun et al., 2015)	- (Sun et al., 2015)			+ (Zhang et al., 2017c)	Up (Li et al., 2017)	Down (He et al., 2017)	Yes (Jiang Y. et al., 2019)
7	Down (Bhaskaran et al., 2019)		- (Babae et al., 2014)	- (Shukla et al., 2018; Yin et al., 2019)	- (Shukla et al., 2018; Yin et al., 2019)	+ (Zhang et al., 2017b; Bhere et al., 2018)	Down (Zhou et al., 2016; McMillan et al., 2017)	Up (Fernández-de Frutos et al., 2019)	Yes (Hu et al., 2019)
338	Down (Han et al., 2017)			- (Klenotic et al., 2004; Lei et al., 2017)	+ (Klenotic et al., 2004; Lei et al., 2017)	- (Klenotic et al., 2004; Lei et al., 2017)	*	Down (Qian et al., 2019)	Yes (Lugli et al., 2015)

miRNAs transported by EVs in these diseases or other types of cancers. *Not described in this work/unknown regulation. Up, upregulation; Down, downregulation. miRNAs transported by EVs in these diseases or other types of cancers.

### miRNAs Associated With Angiogenesis and Proliferation in GBM

One of the most upregulated miRNAs in GBM samples is miRNA27a (Bhaskaran et al., 2019), which belongs to the miRNA-23a–27a–24-2 cluster (Wang N. et al., 2019). miRNA27a regulates proliferation and tumorigenesis in GBM by inhibiting the FOXO3a gene expression (Ge et al., 2013). Accordingly, the administration of an antagonist for miRNA-27a using a lentivirus carrier inhibits GBM proliferation *in vitro* (Feng et al., 2012). Furthermore, miRNA-27a was proposed as a potential marker to distinguish low-grade gliomas from GBM (Rivera-Díaz et al., 2015). Furthermore, miRNA-27a, miRNA-9 (which is known to regulate angiogenesis and proliferation; Yu et al., 2018), and miRNA-23a, were differentially expressed in mesenchymal subtypes compared with proneural subtypes, indicating these three miRNAs might allow distinguishing between GBM molecular subtypes. In addition, these miRNAs segregate between groups of patients with increased and decreased survival rates, suggesting a possible stratification strategy to sort the patients in groups according to high survival and low survival rates by using the three miRNA panel (Marziali et al., 2017).

miRNA-139 is a tumor suppressor, downregulated in GBM (Bhaskaran et al., 2019), which regulates diverse aspects of tumorigenesis such as proliferation, apoptosis, migration, and invasion. The low expression of miRNA-139 correlates with poor prognosis and survival (Chen et al., 2018; Tian et al., 2019). One work has provided evidence that a target of miRNA-139, called NIN1 (RPN12) Binding Protein 1 Homolog (NOB1), which encodes a subunit of the proteasome frequently deregulated in several tumoral processes, is upregulated and correlated with increased metastasis (Shi et al., 2019). Another study has identified several other targets of miRNA-139 in GBM: latrophilin, a seven transmembrane domain containing (ELTD) 1, which encodes an orphan G protein-coupled receptor, and cell cycle progression molecules, such as cyclin A and cyclin D1, supporting its role as a regulator of GBM proliferation (Dai et al., 2015). In addition, the EMT inducers zinc finger E-box-binding homeobox (ZEB) 1 and ZEB2, involved in tumor metastasis, were also identified as targets of miRNA-139. The overexpression of this miRNA causes inhibition of migration and invasion in GBM cell lines likely by downregulating ZEB1 and ZEB2 (Yue et al., 2015). A recent study has also identified syntenin as a downstream target of miRNA-139 in glioma cells. In this context, inhibition of syntenin or miRNA-139 overexpression led to a decreased proliferation, migration, and invasion, and cell cycle arrest confirming previous studies (Tian et al., 2019). Interestingly, induced myeloid leukemia cell differentiation protein (Mcl)-1, an antiapoptotic molecule, also identified as a target of miRNA-139, was able to sensitize GBM cells to temozolomide (TMZ) by modifying Mcl-1 expression (Li et al., 2013).

miRNA-137 is downregulated in GBM (Bhaskaran et al., 2019) and associated with the inhibition of proliferation and angiogenesis. Enhancer of zeste homolog (EZH) 2, a histone-lysine *N*-methyltransferase enzyme involved mainly in the cell cycle, is a target of miRNA-137 and therefore the inhibition of this miRNA led to increased proliferation and angiogenesis, pointing at miRNA-137 as a tumor suppressor (Sun et al., 2015). Another study revealed that miRNA-137 overexpression, by targeting EGFR, led to decreased proliferation and increased apoptosis (Zhang et al., 2017c), further suggesting that low expression of miRNA-137 is correlated with poor prognosis. In addition, miRNA-137 targets genes involved in both neurogenesis and tumorigenesis in GBM, such as c-KIT, CDK6, AKT2, CDC42, YBX1, and transforming growth factor (TGF) β2 (Tamim et al., 2014). Furthermore, these targets were shared with miRNAs −7, −124, and −128, all of these miRNAs are tumor suppressors that will be discussed in the following sections. In addition, miRNA-137 (among other tumor suppressor miRNAs) also targets Musashi-1 expression, a self-renewal and tumorigenic factor in GBM cells, indicating its protective role in this type of tumors (Vo et al., 2011; Velasco et al., 2019). Indeed, based on their opposite expression patterns and function in glioma cells, where they share a common network of target genes, it was further suggested that the balance between miRNA expression could have a critical impact on the dynamics of cellular states affecting tumorigenesis and NDDs development. In addition, miRNA-137 was also reported to regulate mitochondrial dynamics and biogenesis in human induced neural stem cells (hiNSCs) to promote neuronal differentiation. In these cells, miRNA-137 increases the expression of stem cell markers: OCT4 and SOX2, as these molecules have a binding site for miRNA-137 in the promoter (Channakkar et al., 2020). Further exploration of this regulatory network and the role of these master regulators in the modulation of NSC fate would be critical for the understanding of neural maturity, neurological diseases, and GBM cell plasticity and development.

miRNA-15b is upregulated in GBM to induce cell cycle arrest by targeting CCNE1, a protein related to G1/S phase progression (Xia et al., 2009; Bhaskaran et al., 2019). However, other authors found it downregulated in GBM cells and tissues, demonstrating a tumor suppressor function via the targeting of IGF1R, an anti-apoptotic gene (Wang et al., 2017; Matos et al., 2018). Therefore, miRNA-15b low expression is correlated with poor survival (Wang et al., 2017). Moreover, miRNA-15b and miRNA-21 were studied as possible biomarkers in blood samples to distinguish between patients with or without gliomas (Ivo D’Urso et al., 2015). Finally, miRNA-15b levels are decreased in sera from patients with malignant astrocytomas (Jin et al., 2017).

### EVs Containing miRNAs in GBM Angiogenesis and Proliferation

Recent studies addressing the connection between exosomal miRNAs and GBM have begun to disclose many processes of its biology. However, small information is available for miRNAs traveling by GBM exosomes. As an example, miRNA-9 transported by glioma-derived exosomes is upregulated by MYC and OCT4 in GBM cells and causes the dysfunction of the Hypoxia-Inducible Factor (HIF)-1α/VEGF signaling pathway (Chen et al., 2019). miRNA27a is also characterized as a regulator of vital processes in tumor development, including angiogenesis, migration, invasion, proliferation, and apoptosis, and even in tumor resistance and sensitivity for drugs in many types of cancer (Li X. et al., 2016). Detection of miRNA-137 in sera from GBM patients also determined that its low content was correlated with poor prognosis, indicating miRNA-137 could be a prognosis marker (Li X. et al., 2016). Furthermore, miRNA-137 was studied in exosomes related to PD, which will be discussed in the next section (Jiang Y. et al., 2019).

### miRNAs Associated With Migration and Invasion in GBM

Tumor migration and invasion are the major contributors to poor prognosis and relapse. miRNA-21 is highly upregulated in tumor cells, mainly in perivascular niches, and promotes migration and invasion through the β-catenin pathway (Luo et al., 2017). In addition, miRNA-21 might control proliferation modulating the p53 pathway and by interacting with CASC2 long-non-coding (lnc) RNA (Jesionek-kupnicka et al., 2017). Interestingly, Sox2 (stem cell marker) and miRNA-21 are expressed in mutually exclusive patterns in GBM (i.e., high miRNA-21/low Sox2 and low miRNA-21/high Sox2), indicating the existence of two populations of GBM patients with distinct phenotypic features and clinical outcomes. The low miRNA-21/high Sox2 pattern is associated with a better prognosis, consenting a new classification to predict patient survival (Sathyan et al., 2015). Besides, miRNA-21 is one of the miRNAs whose overexpression leads to radioresistance in GBM (Toraih et al., 2019).

miRNA-23a is upregulated in GBM samples according to the TCGA database (Bhaskaran et al., 2019) and was recently identified as a key modulator of GBM invasion by targeting Homeobox 10 and increasing ras homolog family member (Rho)A, RhoC, and urokinase-type plasminogen activator receptor (uPAR) expression (Hu et al., 2013; Yachi et al., 2018). In addition, glial-mesenchymal transition markers metalloproteinase (MMP) 2 and MMP9, were deregulated by this miRNA, indicating an enhanced tumorigenesis. Accordingly, miRNA-23a increases tumor growth, migration, and invasiveness by possibly targeting apoptosis protease-activating factor (APAF) 1, which is involved in apoptosis induction (Lian et al., 2013). Given these characteristics, miRNA-23a has been evaluated as a potential GBM marker for diagnosis, along with miRNA-9 and miRNA-27a, to distinguish the mesenchymal from the proneural subtype (Marziali et al., 2017); however, miRNA-23a is not expressed solely in the CNS and consequently, it may not be used for a diagnosis strategy (Ye et al., 2017). In addition, another study provided a comparative analysis of miRNA patterns between GSCs and non-GSCs (Sana et al., 2018). The authors uncovered nine miRNAs upregulated in GSCs compared to non-GSCs, and, among these, miRNA-9 was inversely correlated with patient survival (Sana et al., 2018).

In the normal brain, miRNA-124a is expressed in developing neurons to ensure its correct maturation (Cao et al., 2007), whereas in GBM is severely downregulated and acts as a tumor suppressor by mainly regulating migration and invasion. It targets IQ motif containing GTPase activating protein (IQGAP) 1, laminin c1, and integrin b1 proteins (Fowler et al., 2011; Bhaskaran et al., 2019); in addition, its expression was negatively correlated with the expression of EMT genes including vimentin (VIM), chitinase-like glycoprotein (YKL)-40, Nestin, and Notch2 (Ma et al., 2012).

miRNA-338 regulates migration, invasion, proliferation, and apoptosis of tumor cells and was found to be downregulated in GBM possibly due to hypermethylation of its promoter (Han et al., 2017). Recently, the EGF-containing fibulin-like extracellular matrix protein 1 (EFEMP1), which controls MMPs, and the metalloproteinase inhibitor (TIMP)-3 were identified as targets of miRNA-338 (Klenotic et al., 2004; Lei et al., 2017). The down-regulation of EFEMP1 inhibits migration and apoptosis in miRNA-338 overexpressing GBM cells. However, miRNA-338 was also found to be upregulated in GBM, when compared to lower grades gliomas, and to promote tumor invasiveness by increasing the expression of MMP2 by downregulating t-shirt zinc finger homeobox (TSHZ) 3, a transcription factor involved in development (Li et al., 2018). Furthermore, miRNA-338 targets embryonic pyruvate kinase M2 (PKM2), frequently overexpressed in tumors, preventing the transcriptional activity of β-catenin and leading to decreased tumor growth and increased survival of the animal model (Han et al., 2017). Moreover, it was discovered that miRNA-338 is regulated by LINC00689, a long non-coding RNA, whose expression is incremented in glioma tissue where it promotes glycolysis, migration, proliferation, and invasion (Liu X. et al., 2019).

### EVs Containing miRNAs in GBM Migration and Invasion

Recent evidence indicates that exosomal miRNAs have control over glioma invasion by modulating their downstream targets and facilitating a spatial communication between tumor cells and their surroundings. For example, miRNA-21 is one of the most upregulated miRNAs traveling by EVs in GBM (de Mooij et al., 2020). When an engineered exosome containing miRNA-21 sponge (a competitive inhibitor for the target miRNA; Ebert et al., 2007) is delivered to GBM cells in culture, these exosomes suppress GBM proliferation and increase apoptosis. Furthermore, miRNA-21- loaded sponge exosomes also decreased the tumor volume in rat models (Monfared et al., 2019). In addition, miRNA-21/miRNA-451 shows a very interesting nexus between microglia, glioma, and EVs. These miRNAs, enriched in glioma-derived exosomes, have a great impact on microglia, where they reduce c-Myc expression, a transcription factor regulating several cellular processes including cell cycle and apoptosis, and promote a shift toward an immune suppressor phenotype, supporting glioma progression (Van Der Vos et al., 2016; Guo et al., 2019).

miRNA-124 is another example of miRNA-loaded in exosomes detected in GBM patients, which was correlated with high grade gliomas, and therefore designated as an onco-miRNA (Tabibkhooei et al., 2020). The delivery of anti-miRNA-124 with a nanocarrier was shown to be effective against GBM by reducing proliferation and migration and inducing cell sensitization to TMZ (Jana et al., 2019). miRNA-124a-loaded exosomes from mesenchymal stem cells (MSCs) were also evaluated as a potential therapeutic strategy to treat GBM (Lang et al., 2018; Bajetto et al., 2020). Both *in vivo* and *in vitro* treatment with these exosomes reduced the tumorigenesis, tumor growth, and increased the survival.

Regarding miRNA-338 loaded exosomes, they were characterized in the context of AD and will be discussed in the next section.

### miRNAs Associated With Apoptosis in GBM

miRNAs also participate in the modulation of the apoptosis pathway in GBM. This is the case of miRNA-210, which is upregulated in GBM and decreases apoptosis by targeting ROD1 (Zhang S. et al., 2018); it was also found upregulated in GSC spheroids under acute hypoxia (Rosenberg et al., 2015). miRNA-210 was proposed as a regulator for TMZ therapy resistance (Lee et al., 2015), since this miRNA is downregulated in TMZ-resistant GBM and its upregulation reverses this condition by targeting P4HB chaperone. Controversially, another study revealed that miRNA-210 is downregulated in GBM where modulates migration and invasion by targeting BDNF (Liu S. et al., 2019).

Another example is miRNA-7, which is downregulated in GBM and promotes TRAIL-induced apoptosis (Bhaskaran et al., 2019). Accordingly, forced expression of miRNA-7 and exposure to TRAIL raised the proportion of apoptotic GBM cells (Zhang et al., 2017b).

miRNA-10b is another miRNA highly upregulated in GBM samples (Bhaskaran et al., 2019), but, interestingly, it is not expressed in normal brain tissue (Gabriely et al., 2011). Regarding apoptosis, miRNA-10b targets BIM (Gabriely et al., 2011), a molecule that participates in the mitochondrial cell death pathway, hence preventing apoptosis in GBM. Furthermore, together with miRNA-222, miRNA-10b prevents apoptosis by p53 dependent and independent pathways (Sun et al., 2019): miRNA-10b and miRNA-222 target PTEN, therefore activating MDM2 and inhibiting p53; and also inhibit PUMA and BIM, respectively. Besides, miRNA-10b also targets p21 and E2F1, contributing to deregulation of cell cycle, and promotion of proliferation and tumor growth (Teplyuk et al., 2015). In GBM cells, miRNA-10b was also shown to mediate the effects of and to be upregulated by TGF-β1 (Ma et al., 2017). Furthermore, miRNA-10b increases proliferation, invasion, and EMT by targeting PTEN, caspase-9, Apaf-1, and E-cadherin (Ma et al., 2017). As mentioned above, miRNA-10b is not expressed by normal brain tissue, therefore constituting an interesting candidate for therapeutic approaches.

miRNA-106b is another highly upregulated miRNA in GBM samples. It has been described as an inhibitor of apoptosis and as a proliferation enhancer by targeting retinoblastoma-like 1/2 and CASP8 (Liu et al., 2014). Interestingly, miRNA-106b decreases NKG2D, an activating ligand for NK cells, pointing at an immune suppressor miRNA (Codo et al., 2014).

miRNA-155 is highly expressed not only in GBM (Bhaskaran et al., 2019) but also in a number of other cancers (Bayraktar and Van Roosbroeck, 2018). It promotes proliferation of GBM cells by targeting both MXI1, a c-Myc inhibitor (Zhou et al., 2013) and targets a p38 isoform which promotes apoptosis (Liu et al., 2015). In addition, it modulates invasion by increasing the secretion of MMP2 and MMP9 (Liu et al., 2015). Furthermore, its downregulation sensitizes GBM cells to TMZ and reduces their proliferation (Liu et al., 2015). miRNA-155 is derived from the long non-coding RNA, miRNA-155 hostgene (HG), which has been described mainly as an EMT promoter (Cui et al., 2019). In GBM, miRNA-155HG was also correlated with poor prognosis, malignancy, EMT and tumor progression; these effects were the result of the modulation of miRNA-155 expression and its downstream target genes, such as protocadherin 7 and 9, both acting as tumor suppressors (Wu et al., 2017).

### EVs Containing miRNAs in GBM Apoptosis

miRNA-210 was found in EVs isolated from GBM patient sera showing a significant upregulation when compared with the control group. Therefore, EV-miRNA-210 could eventually be considered as a marker for GBM (Lai et al., 2015).

Even though miRNA-10b has not yet been detected in exosomes from GBM, the delivery of miR-10b antisense oligonucleotide inhibitors to mouse orthotopic xenografts resulted in a slow-down of tumor progression (Teplyuk et al., 2016). Also, the intravenous injections of encapsulated antago-miRNA-10b, together with antago-miRNA-21, in synthesized nanoparticles (Malhotra et al., 2018), showed cell cycle arrest and decreased of tumor volume after TMZ treatment.

miRNA-7 loaded exosomes were detected in TRAIL-overexpressed MSCs transiently expressing miRNA-7, indicating its EV-loading property. When injected into the tail vein of GBM-xenografted mice, these cells promoted GBM cell death and decreased tumor growth (Zhang et al., 2017b). Another study reported that miRNA-7 delivered to GBM by several methods such as adeno-associated virus, induced GBM cell apoptosis by targeting EGFR, linked to upregulation of death receptor 5 and subsequent TRAIL-induced cell death (Bhere et al., 2018). Moreover, miRNA-7 overexpression in GBM cells diminished migration and invasion, targeting SATB1 (Yin et al., 2019). Furthermore, miRNA-7 was also linked to anti-angiogenic effects: its delivery in polymeric nanoparticles to GBM xenografts, diminished tumor growth and decreased angiogenesis (Babae et al., 2014). Even though miRNA-7 containing EVs have not yet been isolated from GBM, this miRNA makes a promising candidate for therapeutic approach, since it targets several physio-pathological processes in GBM.

miRNA-106b was only detected in exosomes derived from sera from patients with low grades gliomas, but not yet from GBM patients (Ebrahimkhani et al., 2018).

## miRNAs as Modulators of Parkinson’s and Alzheimer’s Diseases

**FIGURE 3:**
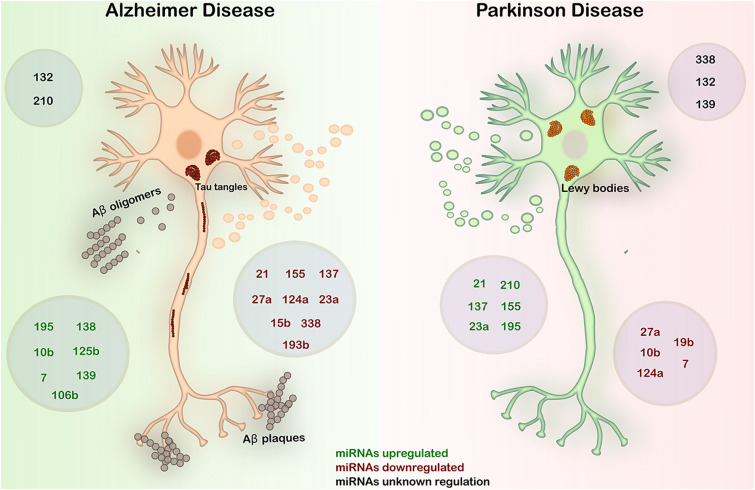
Expression in AD and PD of the most deregulated miRNAs in GBM. AD is characterized by the deposition of Aβ fibrils in the brain parenchyma and vessel walls and by the accumulation of aberrant phosphorylated Tau protein in neurons. PD is an α-synucleinopathy characterized by the formation of Lewy bodies by the accumulation of α-synuclein predominantly in neurons, causing the loss of dopaminergic neurons. In both diseases, abnormal regulation of miRNAs plays a key role in the onset and progression of the diseases. Black – miRNAs with unknown regulation in AD and PD; green – miRNAs upregulated in AD and PD; red – miRNAs downregulated in AD and PD.

In Figure 3 and Table 1, we summarized the expression patterns of miRNAs in AD and PD described in this work. We will focus on these two main NDDs to discuss about the inverse comorbidity with cancer previously described (Driver, 2014).

### miRNAs Modulating Alzheimer’s Disease

The impaired learning and memory characteristic of AD is correlated with increased miRNA-139 levels in the hippocampus of senescence-accelerated mouse-prone (SAMP) 8 mice. This effect was associated with the targeting of cannabinoid receptor type 2, involved in microglia inflammatory responses (Tang et al., 2017). Besides, miRNA-139 decreased pro-inflammatory responses by targeting ICAM-1 and CD40. Overall, it was determined that the inhibition of miRNA-139 with antagomir injection reduced AD pathology. It is important to note that in GBM, miRNA-139 is downregulated and acts as a tumor suppressor. Since this miRNA participates in inflammatory responses, it would be interesting to evaluate its role in this process in GBM.

miRNA-137, which regulates the NF-kB pathway by targeting (TNFα)-induced protein 1 (TNFAIP1), is downregulated in AD. In support, miRNA-137 overexpression decreases Aβ toxicity in neurons by inhibiting the NF-kB pathway (He et al., 2017). Besides, Aβ peptide induces miRNA-137 downregulation in cultured neurons.

A high miRNA-7 level is associated with high Aβ protein deposits in AD. This miRNA targets Insulin receptor, Insulin Receptor Substrate-2, and Insulin Degrading Enzyme, which are critical to glucose homeostasis and Aβ metabolism in AD (Fernández-de Frutos et al., 2019). In addition, miRNA-7 was found directly correlated with an increase of Aβ protein deposition in neurons and decrease clearance by microglial cells (Fernández-de Frutos et al., 2019).

miRNA-132 has a protective effect in AD as it is involved in neuronal plasticity, Tau downregulation, and protection from glutamate excitotoxicity (El Fatimy et al., 2018). Despite this neuroprotective role, miRNA-132 was associated with Tau hyperphosphorylation by targeting glycogen synthase kinase 3β (GSK3β) and sirtuin 1 (SIRT1), responsible for Tau phosphorylation and acetylation, respectively (Dehghani et al., 2018). Other miRNAs also regulate SIRT1 expression, including miRNA-181c and miRNA-9. Another example is miRNA-138, which is upregulated in AD and reduces the expression of SIRT1, and also increases the activation of GSK3β promoting Tau aggregation (Dehghani et al., 2018). Therefore, SIRT1 would be a candidate node that might link between these miRNAs to AD pathogenesis.

miRNA-195 is involved in the formation of Tau aggregates and Aβ plaques by upregulating β-secretase (BACE) 1. It also activates CDK5, which leads to Tau hyperphosphorylation, and nuclear factor kappa B (NFkB) activation, causing neuroinflammation (Dehghani et al., 2018). miRNA-125b also regulates Tau phosphorylation by activating MAPK. It also regulates synapsis by targeting a subunit of *N*-methyl-D-aspartate (NMDA) glutamate receptors (Dehghani et al., 2018).

Recently, downregulated miRNA-23a was detected in sera from AD patients that carry a SNAP-25 genotype, which is correlated with brain atrophy and cognitive decline (Agostini et al., 2019). In addition, deregulated miRNA-23a was found in both AD and PD, and associated to ongoing neurodegenerative processes. miRNA-210 was also found reduced in AD patients sera and cerebro-spinal fluid (CSF), and correlated with diminished VEGF expression, an angiogenic growth factor, and with the severity of the disease (Zhu et al., 2015; Swarbrick et al., 2019).

miRNA-124a is implicated in AD pathogenesis because it downregulates hypoxia- associated Aβ formation through the disinhibition of BACE1 (Zhang et al., 2017a). This is an important finding that would corroborate the theory of vascular dysfunction and hypoperfusion during the pathogenesis of AD (Govindpani et al., 2019). Similarly, overexpressed miRNA-338 targets BACE1 and reverts neuroinflammation and Aβ-amyloid formation (Qian et al., 2019). Interestingly, the knockdown of this miRNA by the injection of the miRNA-338-sponge in mice brain promoted spontaneous neoplasm formation, histologically reminiscent of GBM with neuronal morphological disorganization and increased proliferation (Howe et al., 2017). This data is of utmost importance as miRNA-338 is severely downregulated in GBM (Han et al., 2017), and the solely fact that its downregulation promotes neoplasm formation indicates that it is a key player for developing brain tumors. Besides, miRNA-338 is highly expressed in mature neurons, and its overexpression in GBM cell lines decreased their proliferation (Howe et al., 2017). Worth to notice, both miRNA-124a and −338 target BACE1 and are upregulated in AD and downregulated in GBM. In this regard, Aβ peptide accumulation has been found in GBM samples from patients and mouse models (Zayas-Santiago et al., 2020), mainly in glioma blood vessels walls and in GBM cells that expressed GFAP. As miRNA-124a and −338 promote vascular dysfunction and inflammation, it would be interesting to know if these miRNAs are involved in Aβ accumulation in GBM.

Likewise, a search for common miRNAs between cancer and AD allowed the identification, in both diseases, of a common expression of miRNA-27a, miRNA-7 and miRNA-21, all targeting EGFR (Battaglia et al., 2019). Despite that GBM was not evaluated in this work, all of these miRNAs are important as we described above. Other miRNAs shared by AD and GBM are miRNAs-9, 106b, −124, −132 and 138, all related to Tau phosphorylation. In addition, miRNAs-9 and 132 were also linked to other Tau post-translational modifications, such as acetylation (Bazrgar et al., 2020). In addition, miRNA-9, −124, −132 and −137, among others were related to Tau alternative splicing.

### EVs-miRNAs in Alzheimer’s Disease

Recently, one revision of exosomes modulating AD has classified EVs-miRNAs according to the biological fluids collected for the study (Martins et al., 2020). They observed that miRNAs in serum-derived EVs were often upregulated, whereas in plasma, were mostly downregulated. From the miRNAs found in serum-derived EVs, it is worth to point out that miRNA-23a and miRNA-106b were upregulated, and both target key enzymes of APP and Tau metabolism. Both miRNAs are also upregulated in GBM (Bhaskaran et al., 2019), where miRNA-23a modulates mainly migration and miRNA-106b regulates apoptosis and proliferation.

miRNA-15b and 193b are downregulated in serum-derived EVs from AD patients, yet their targets in AD are unknown. By contrast, in GBM, miRNA-15b is upregulated and regulates cell cycle, whereas miRNA-193b is poorly described: only one study found it upregulated in pericytes of GBM microvasculature, where it modulates the expression of PDGFRβ and regulates proliferation (Xu and Li, 2016). These miRNAs were not identified in GBM exosomes yet, nonetheless, results from AD EVs are promising to be further exploited in GBM.

In plasma-derived EVs, miRNA-21, miRNA-23a, miRNA-132, miRNA-193b, and miRNA-338 are downregulated (Martins et al., 2020). Notably, miRNA-23a shows an opposite accumulation between serum and plasma. Among these miRNAs, they are all upregulated also in GBM, except miRNA-338, which is downregulated (Bhaskaran et al., 2019). miRNA-21 is downregulated in AD (Juźwik et al., 2019); however, one work has shown that miRNA-21-containing exosomes derived from SH-SY5Y neuroblastoma cells transfected with the Swedish mutant of APP_695_ (SHSwe), induce microglia M1 polarization, which releases pro-inflammatory cytokines such as high mobility group box 1 protein (HMGB1), tumor necrosis factor (TNF)-α, S100 calcium-binding protein B (S100B), and further secretes miRNA-21-containing exosomes, probably as a positive feedback mechanism (Fernandes et al., 2018). These facts led to consider miRNA-21 as a potential enhancer of AD pathogenesis since the pro-inflammatory M1 polarized microglia downregulate genes involved in Aβ-protein clearance and thus promotes the accumulation of oligomers and disease progression (Hickman et al., 2008). On the contrary, another work described that the increase of miRNA-21 content in an AD mouse model reduced the cognitive deficit, diminished the Aβ-deposition, and decreased inflammatory markers (Cui et al., 2018). The artificial increase of miRNA-21 was achieved by the administration of exosomes from hypoxia-preconditioned MSCs.

In CSF of AD patients, miRNA-27a and miRNA-132 are upregulated and miRNA-193b is downregulated (Martins et al., 2020). Another work found that reduced miRNA-27a content in CSF was associated to elevated Tau and reduced Aβ-amyloid CSF levels (Frigerio et al., 2013).

Another recent study analyzed EV-miRNAs from AD patient plasma, and compared miRNAs derived from total EVs with those from neural origin (Serpente et al., 2020). Thus, neural-derived EVs (NEVs) were isolated by selecting the EVs expressing LCAM1. They found that miRNA-23a was present and upregulated in both EVs and NEVs, though miRNA-15b and miRNA-27a were upregulated in total EVs, but not in NEVs. These results support the hypothesis that these miRNAs are dysregulated in both AD and GBM.

A recent study analyzed the content of EVs from AD, Lewy bodies dementia (also characterized by the presence of Aβ-amyloid plaques and hyperphosphorylated Tau) and healthy patients. The analysis of plasma-derived EVs, determined that miRNA-21 and miRNA-451 allowed the discrimination between AD and dementia with Lewy bodies, and therefore could represent a future diagnosis strategy (Gámez-Valero et al., 2019). In GBM, EVs containing miRNA-21 and −451 shift microglia to an immune suppressor phenotype, as described above (Van Der Vos et al., 2016; Guo et al., 2019), hence it would be interesting to know if this pair of miRNA produces the same effects in AD, since it has a major inflammatory component. Furthermore, a set of four miRNAs (23a, 126, let-7i, and 151a) were decreased in EVs from AD compared to control. These results are in disagreement with previously discussed studies, stating that miRNA23a is upregulated. Altogether, there is still much controversy regarding EVs cargo analysis, and these will stay as long as a standardized method is developed and validated.

### miRNAs Modulating Parkinson’s Disease

In PD, the upregulation of miRNA-21 induces α-synuclein expression, indicating a detrimental role of this miRNA (Su et al., 2016). This upregulation contrasts with the downregulation described in AD (Juźwik et al., 2019).

Recent studies using cell lines treated with the neurotoxin 1-methyl-4-phenylpyridinium (MMP+), which *in vivo* reproduces the same neurodegeneration pattern observed in PD patients, induced the upregulation of miRNA-210, which correlated with poor disease outcome and dopaminergic neuron damage by targeting BDNF (Zhang S. et al., 2018). This factor is neuroprotective, improving neuronal survival, and synaptic function and plasticity (Winham et al., 2019); conversely, in GBM, BDNF promotes tumor cell stemness (Wang X. et al., 2018). This inverse relation would need further studies since the same miRNA targeting the same factor produces different outcomes.

miRNA-124a is also decreased in PD patients, but, when delivered to the subventricular zone (SVZ) with encapsulating nanoparticles in a 6-hydroxydopamine (6-OHDA) mouse model for PD, promotes improvement of motor symptoms (Saraiva et al., 2016). The family of miRNA-124 has been deeply studied in PD as it targets several neuroprotective pathways and molecules: STAT-3, calpain-1, Bim, Annexin V, and MEKK3. In GBM, miRNA-124a is also severely downregulated and acts as a tumor suppressor by modifying mainly migration and invasion, although there is no common known target with PD (Fowler et al., 2011; Bhaskaran et al., 2019).

miRNA-137 is upregulated in patients with PD, nevertheless no correlation was found between this increase and the severity of the disease (Li et al., 2017); furthermore, miRNA-137 is also elevated in plasma and could represent a candidate biomarker for PD (Li et al., 2017). As discussed above, miRNA-137 is downregulated in GBM and acts as a tumor suppressor, and targets EZH2 to modulate proliferation and angiogenesis (Sun et al., 2015). Since epigenetic dysregulation accompanied by cognitive dysfunction is a common feature in many neurological disorders, it would be interesting to know the epigenetic status regarding EZH2 activity in PD and AD patients.

Downregulation of miRNA-7 promotes α-synuclein accumulation contributing to the loss of dopaminergic neurons (Zhou et al., 2016; McMillan et al., 2017). Likewise, it was recently reported that this miRNA promotes the degradation of α-synuclein by autophagy (Kovacs, 2017). Besides, miRNA-7 targets NOD-, LRR-, and pyrin domain-containing protein (NLRP) 3, a component of inflammasomes that is increased in PD patients and PD-mice models (Zhou et al., 2016). Moreover, miRNA-7 injection into the brain of a PD-mice model promotes the disassembly of the inflammasomes, decreasing the neuroinflammation, and protecting the dopaminergic neurons from degeneration. Furthermore, miRNA-7 overexpression protected cortical neurons from MPP+-induced toxicity, favoring neuronal viability, and preventing apoptosis (Fragkouli and Doxakis, 2014). In GBM, miRNA-7 is downregulated and controls apoptosis (Zhang et al., 2017b), whereas in PD, it regulates α-synuclein degradation and inflammation (Kovacs, 2017).

### EVs-miRNAs in Parkinson’s Disease

In PD, compared to other NDDs, only a few studies described the pathogenetic role of EVs-miRNAs. One study evaluated miRNA-27a as a possible biomarker in CSF-exosomes (dos Santos et al., 2018), similar to AD. Another study reported that mice serum exosomes enriched with miRNA-10b generated M2 polarization, indicative of the induction of an anti-inflammatory phenotype (Zhou et al., 2017). The level of miRNA-10b is decreased and positively correlated with age of onset of PD (Hoss et al., 2016). It would be interesting to determine the targets affected by miRNA-10b to promote M2 polarization in PD and the ones that promote apoptosis in GBM to deep in the molecular causes and possible therapeutics for these diseases.

It was demonstrated that miRNA-124a is transferred from neurons to astrocytes via exosomes and this interaction promotes the expression of GLT1, a glutamate transporter (Morel et al., 2013). Another work determined that miRNA-137 containing sera-derived exosomes led to oxidative stress injury in neurons by targeting OXR1, while exosomes carrying miRNA-137 antagomir alleviate the symptoms, representing a possible therapeutic approach (Jiang Y. et al., 2019).

A recent review collected literature regarding EVs-miRNAs in PD (Wang and Zhang, 2020). Among the miRNAs we analyzed, elevated miRNA-23a and miRNA-195 were found in serum-derived exosomes, like in AD, while miRNA-19b is downregulated both in serum- and in CSF-derived exosomes. The targets of miRNA-19b and −195 miRNA have not been yet elucidated in PD. However, as far as miRNA-19b is concerned, *in silico* GO analysis determined that its targets are involved in macromolecule biosynthetic process (Uwatoko et al., 2019). Another recent work demonstrated that miRNA-21, miRNA-210, and miRNA-155 are present in plasma-derived EVs from a pre-motor PD model, with the total number of EVs also incremented (Sancandi et al., 2020). As discussed above, miRNA-21 represents a common dysregulated EV-miRNA among all the diseases studied, where it fulfills different functions. For example, in the MMP+ PD cell line model, miRNA-21 was increased, and the treatment with miRNA-21 inhibitor reduced apoptosis, improved survival and inhibited the production of pro-inflammatory cytokines (Mao and Ding, 2019); in AD, when traveling by EVs, it also modulates microglia polarization toward an M1 phenotype (Hickman et al., 2008), whereas in GBM, it promotes an immune suppressor shift in microglia (Van Der Vos et al., 2016; Guo et al., 2019). The main shared aspect of this miRNA function in the different diseases studied, is microglia polarization, thus it would be interesting to further explore the targets affected by this miRNA in this process as a possible therapy to be developed.

miRNA-210 was found upregulated in MMP+ PD model in SH-SY5Y cells, in which, targeting BDNF, contributes to cell damage (Zhang S. et al., 2018). Interestingly, miRNA-210 targets BDNF in both PD/AD and GBM, and as we described above, it is a neuroprotective factor in AD and PD, and protumoral in GBM. It has not yet been found in exosomes from GBM or AD, nevertheless, it is present in CSF and serum from AD patients, so further research for this miRNA would be interesting to develop possible future therapies. miRNA-155 level is increased in an *in vivo* model of PD in which α-synuclein expression is induced by adenovirus inoculation and, similarly to AD (Slota and Booth, 2019), was markedly linked to inflammation (Thome et al., 2016), whereas in GBM it was related to cell proliferation, apoptosis and migration. However, further research for the role in inflammation needs to be addressed as it has not yet been described in GBM.

## Conclusion

Here we describe and discuss the molecular pathways shared by GBM and NDDs, as an approach to explaining the epidemiological mutual exclusive comorbidities, while analyzing classical pathogenic mechanisms would be too difficult. Yet, a no clear correlation of miRNA expression pattern observed herein GBM and NDDs. However, a similar approach dealing with these inverse comorbidities between cancer and NDDs found that specific pathways such as WNT, p53, protein folding, and protein degradation are inversely regulated (Ibáñez et al., 2014). Indeed, from our analysis, some miRNAs inversely expressed and targeting the identical molecule or modulate the same pathway in both GBM and NDDs would represent attractive entry points to a deeper understanding of the underlying molecular physiopathological mechanism. For example, miRNA-210 targeting BDNF, miRNA-21 modulating microglia, and miRNAs −27a and −132 modulating Tau would contribute to enlightening what pathway triggers a neuron to transform into an undifferentiated and immortal tumor cell or deteriorated dying cells. It is also worth noticing that miRNA-10b does not express in normal brain tissue (Gabriely et al., 2011), so it would represent an attractive diagnostic approach.

Regarding NDDs, in addition to mutations found in the pathognomic proteins, specific miRNAs expressions are significantly altered during disease progression, proposing them as contributing factors of these diseases (Figure 3 and Table 1), despite the molecular and cellular functions of these miRNAs are poorly studied.

GBM is a very aggressive cancer characterized by a very low rate of patients’ 5-years survival. Part of the problem is because the symptom’s appearance and the tumor detection are often belated; consequently, the tumor reaches larger dimensions than treatable. Also, the highly cellular plasticity that drives these tumors with a massive spreading within brain parenchyma and their heterogeneous microenvironment contributes to the difficult scenario. Current therapy is ineffective, being the surgery the most appropriate treatment; however, given its high degree of invasiveness, the removal of the tumor is usually incomplete and the tumor relapses. Although its early detection is critical, there is not a currently available method. Therefore, deregulated expression of specific miRNAs, some of them found regularly loaded in EVs and associated with cancer progression, could be used as biomarkers and therapy design. Indeed, some of them, and also other entities in liquid biopsies such as cell-free DNA, are currently being evaluated to provide accurate and complementary information about the molecular or genetic makeup of this heterogeneous tumor. However, the development of new technology is required to advance in this aspect.

Further efforts should be done to better understand the complexity of these pathologies; by deeply exploring the diversity of deregulated miRNAs network and their potential association with EVs. This is an important aspect to comprehend their regulatory action, their roles in the major pathways, and to find potential treatments. This review intends to provide the most recent updates in the field to explore the potential nexus between GBM and NDDs, by discussing the major pathophysiological regulatory points, focusing on those deregulated miRNAs, mostly associated with EVs. Therefore, we consider we have provided a comprehensive insight into the molecular mechanisms, to find common and opposite elements to be considered in future research.

## Author Contributions

LT wrote, discussed, corrected the manuscript, and performed the figures. TF discussed and corrected the manuscript. CP-C designed the study, discussed and corrected the manuscript. All authors contributed to the article and approved the submitted version.

## Conflict of Interest

The authors declare that the research was conducted in the absence of any commercial or financial relationships that could be construed as a potential conflict of interest.

## Footnotes

1 http://www.exocarta.org/

2 http://www.mirbase.org/

3 https://portal.gdc.cancer.gov/projects/TCGA-GBM/

4 https://www.ncbi.nlm.nih.gov/gds

5 http://bigd.big.ac.cn/gliomaDB

## Abbreviations

AD: Alzheimer’s disease
AKT2: RAC-beta serine/threonine-protein kinase
APP: amyloid precursor protein
BBB: blood–brain barrier
CD: cluster of differentiation
CDC42: cell division control protein 42
CNS: central nervous system
DNA: deoxyribonucleic acid
EMT: epithelial–mesenchymal transition
Epi-miRNA: epigenetic miRNA
EV: extracellular vesicle
FOXO: forkhead box O
GBM: glioblastoma
GSCs: GBM stem cells
IDH: isocitrate dehydrogenase
KANSL2: KAT8 Regulatory NSL Complex Subunit 2
MAPK: mitogen-activated protein kinase
NDDs: neurodegenerative diseases
NSCs: neural stem cells
OCT4: octamer-binding transcription factor 4
OXR1: oxidation resistance 1
PD: Parkinson’s disease
PDGFRA: platelet-derived growth factor receptor alpha
POUxFx: Pit-Oct-Unc Factor
RNA: ribonucleic acid
SALL2: Spalt Like Transcription Factor 2
Sox: Sex determining Region Y-box
TMZ: Temozolomide
VFGF: vascular endothelial growth factor
YBX1: Y-Box Binding Protein 1.
